# Monitoring cerebral hemodynamic change during transcranial ultrasound stimulation using optical intrinsic signal imaging

**DOI:** 10.1038/s41598-017-13572-0

**Published:** 2017-10-13

**Authors:** Evgenii Kim, Eloise Anguluan, Jae Gwan Kim

**Affiliations:** 1 School of Electrical Engineering and Computer Science, Gwangju Institute of Science and Technology, Gwangju, 61005 Korea; 2 Department of Biomedical Science and Engineering, Gwangju Institute of Science and Technology, Gwangju, 61005 Korea

## Abstract

Transcranial ultrasound stimulation (tUS) is a promising non-invasive approach to modulate brain circuits. The application is gaining popularity, however the full effect of ultrasound stimulation is still unclear and further investigation is needed. This study aims to apply optical intrinsic signal imaging (OISI) for the first time, to simultaneously monitor the wide-field cerebral hemodynamic change during tUS on awake animal with high spatial and temporal resolution. Three stimulation paradigms were delivered using a single-element focused transducer operating at 425 kHz in pulsed mode having the same intensity (I_SPPA_ = 1.84 W/cm^2^, I_SPTA_ = 129 mW/cm^2^) but varying pulse repetition frequencies (PRF). The results indicate a concurrent hemodynamic change occurring with all actual tUS but not under a sham stimulation. The stimulation initiated the increase of oxygenated hemoglobin (HbO) and decrease of deoxygenated hemoglobin (RHb). A statistically significant difference (p < 0.05) was found in the amplitude change of hemodynamics evoked by varying PRF. Moreover, the acoustic stimulation was able to trigger a global as well as local cerebral hemodynamic alteration in the mouse cortex. Thus, the implementation of OISI offers the possibility of directly investigating brain response in an awake animal during tUS through cerebral hemodynamic change.

## Introduction

Brain stimulation plays an important role in both general neuroscience as well as in clinical applications^1–3^. It has been shown to be an effective therapeutic application for treating neurological disorders including Parkinson’s disease^4^, major depressive disorder^5^, Tourette syndrome^6^, and obsessive-compulsive disorder^7^, among others^8^. However, most current stimulation approaches suffer from shortcomings: (*i*) transcranial direct current stimulation and (*ii*) transcranial magnetic stimulation are both powerful, non-invasive modalities to innervate cortical neurons that are, however, limited by spatial specificity^9^; (*iii*) deep-brain stimulation, although able to provide a higher spatial specificity, requires surgical intervention^10^; (*iv*) and lastly, optogenetics offers even superior targets of single neuron excitation, but involves genetic modification^11^. On the other hand, low intensity ultrasound stimulation (US) offers a non-invasive approach to stimulate inside the brain with spatial resolutions of up to several mm.

Previous studies have shown the ability of US to modulate the neural tissue, both *ex vivo* ^12,13^ and *in vivo* ^14,15^, for animal^16^ and human subjects^17,18^. Ultrasound has been demonstrated to reversibly inhibit visual evoked potentials in cats^19^ and excite the motor cortex of rats prompting a tail motor response^20^ depending on acoustic pulse parameters. The acoustic stimulation has also been found to increase extracellular levels of serotonin and dopamine when targeting the rat thalamus^21^. In addition, sonication targeted at the human sensory cortex was able to elicit tactile sensation on the subject’s hand area contralateral to the stimulated hemisphere^22^. More recently, transcranial ultrasound stimulation (tUS) has been demonstrated to be a powerful technique in clinical application by facilitating the recovery of a patient from coma after severe brain injury using thalamic ultrasound-based stimulation^23^. While low intensity tUS is considered safe and has already been implemented for human participants, pre-clinical studies that aim to determine the effects of tUS on the brain still need to be carried out.

Several studies have been conducted to measure the effect of tUS by simultaneous recording of electrical activity in the brain using electroencephalogram (EEG)^24,25^, multi-unit activity (MUA)^26^ and local field potential (LFP)^27^, as well as in muscle using electromyography (EMG)^28^. These approaches provide a direct measure of neural response to US with superior temporal resolution but are unable to offer a broader view of brain activity at high spatial resolution. Whole brain functionality as influenced by tUS has been observed in combination with functional magnetic resonance imaging (fMRI) and positron emission tomography (PET). Functional MRI studies reveal that tUS excites not only the sonicated region in the human brain, but also other network related regions^29,30^; while PET on an anaesthetized rat have shown the increase of glucose consumption in the brain is smaller than the sonicated region^31^. Although fMRI and PET provides 3D information, they are limited by the high cost, bulky size, low temporal resolution and the complexity of implementation particularly for animal models without anaesthesia. Anaesthesia has been shown to significantly alter brain function^32–34^, and the importance of anaesthesia level has been emphasized in many ultrasound stimulation studies showing the necessity of a light anaesthesia state to elicit a motor response during brain stimulation^35,36^. Thus, we propose to apply optical intrinsic signal imaging (OISI) on an awake mouse model as a low cost, compact, simple neuroimaging technique to provide concurrent mesoscopic functional information during ultrasound brain stimulation.

The application of OISI in brain studies was driven by the similarity in contrast mechanism with fMRI which primarily uses the blood-oxygen level dependent (BOLD) signal as an indicator of neural activity through the hemodynamic response function (HRF). In a similar fashion, OISI relies on the distinct light absorption spectra of oxy- and deoxyhemoglobin, providing the relative change in oxygenated (HbO) and deoxygenated (RHb) hemoglobin concentration. Monitoring the change in HbO and RHb offers an indirect way of recording brain activity based on the neurovascular coupling mechanism^37^. Although, OISI may not provide deep brain information, it is an attractive and accessible approach to imaging large areas of the brain with high spatial and temporal resolution. The OISI technique has been used in various neuroscience studies to investigate functional brain connectivity^38,39^, anaesthetic effect on the brain^32^, as well as to explore other neuromodulation approaches^40,41^.

## Results

### Cerebral hemodynamic change during tUS

**Figure 1:**
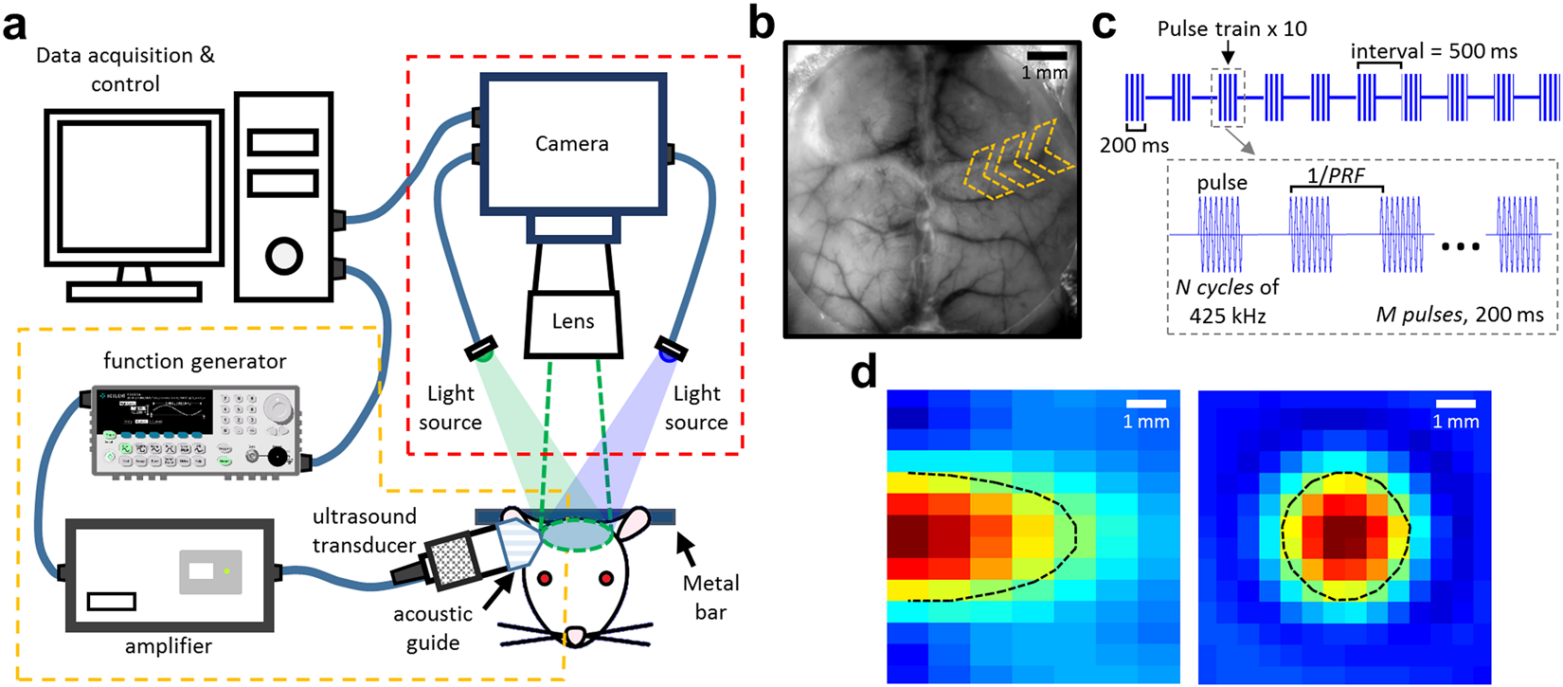
Schematic of the setup for monitoring the cerebral hemodynamics during tUS. (**a**) The schematic diagram of the setup as implemented with an awake mouse restrained on a stereotaxic frame by means of a metal bar attached to the head. Stimulation was performed using a single-element ultrasound transducer driven by a pulse-modulated amplified waveform from a function generator. The transducer was fitted with an acoustic guide and placed on the right side of the mouse head. Image acquisition was achieved using an sCMOS camera with two light sources (465, 560 nm) alternately illuminating the brain during stimulation at a rate of 33 Hz each. (**b**) An image of the mouse cortex through the cranial window under 560 nm illumination. The arrow indicates the direction of the ultrasound beam. (**c**) Acoustic parameters for the tUS. The ultrasound delivered was a 425 kHz pulse-modulated signal containing 10 pulse trains given over a period of 5 seconds for each trial. Every stimulus is a pulse train lasting 200 ms with *M* pulses given at a rate determined by the pulse repetition frequency (PRF), with each pulse having *N* cycles. (**d**) Longitudinal and transverse views of the ultrasound beam profile measured 2 mm from the edge of the acoustic guide. The focal length of the transducer extends 7.5 mm longitudinally and the focal size is 3 mm in diameter, as determined by the FWHM of the acoustic intensity (black dashed contour).

A total of seven awake mice were used to explore the tUS effect on cerebral hemodynamics using OISI (Fig. 1). An intact skull cranial window provided a minimally invasive view of the mouse cortex during stimulation. The spatiotemporal hemodynamic change was monitored under three different tUS conditions (Table 1) and sham.

**Table 1:** US parameters.

#	Central frequency (kHz)	Pulse Repetition Frequency (Hz)	N cycles	M pulses	I_SPPA_ (W/cm^2^)	I_SPTA_ (mW/cm^2^)	Negative Pressure (MPa)	Mechanical Index
1	425 kHz	375	80	75	1.84	129	0.53	0.81
2		750	40	150				
3		1500	20	300				

**Figure 2:**
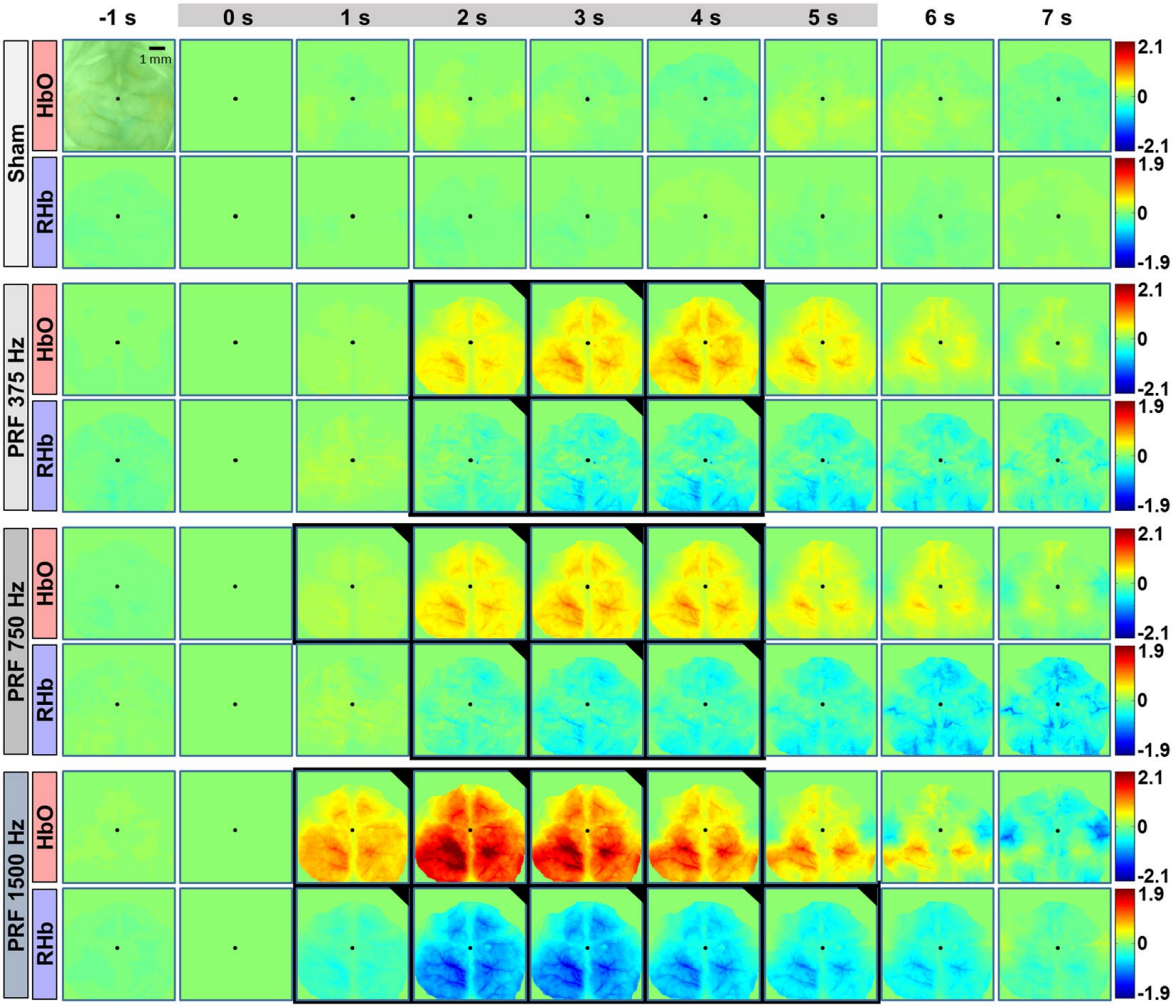
Spatial map of the hemodynamic changes for the different tUS conditions in a representative mouse. Stimulation occurred during the frames for 0–5 s. The baseline image was taken at 0 s, at the onset of stimulation. The color bars indicate the concentration change in µM. The frames with black borders indicate time points for which there is a statistically significant difference (one-way ANOVA with Tukey-Kramer post-hoc analysis; p < 0.05) in the hemodynamic change compared to sham. Overlaid on the -1 s frame for HbO of sham is an image of the mouse cortex at 560 nm for reference. The black dots indicate the bregma. A video of the hemodynamic change for PRF 1500 Hz is available as Video 1.

The stimulation was confirmed to elicit a cerebral hemodynamic change for the three actual tUS conditions but not in sham. Figure 2 shows the spatial map of propagation of filtered HbO and RHb over time in a representative mouse (Video 1). The frames labelled 0 and 5 s correspond to the hemodynamic change at the beginning and end of the stimulation, respectively. The three sonication paradigms applied have different PRF but with constant acoustic power. All of which evoked a statistically significant different hemodynamic change compare to the sham stimulation (one-way ANOVA with Tukey–Kramer *post-hoc* analysis, *p* < 0.05), specifically the increase of HbO and decrease of RHb. A similar spatial distribution of hemodynamics was observed from all mice in the study.

**Figure 3:**
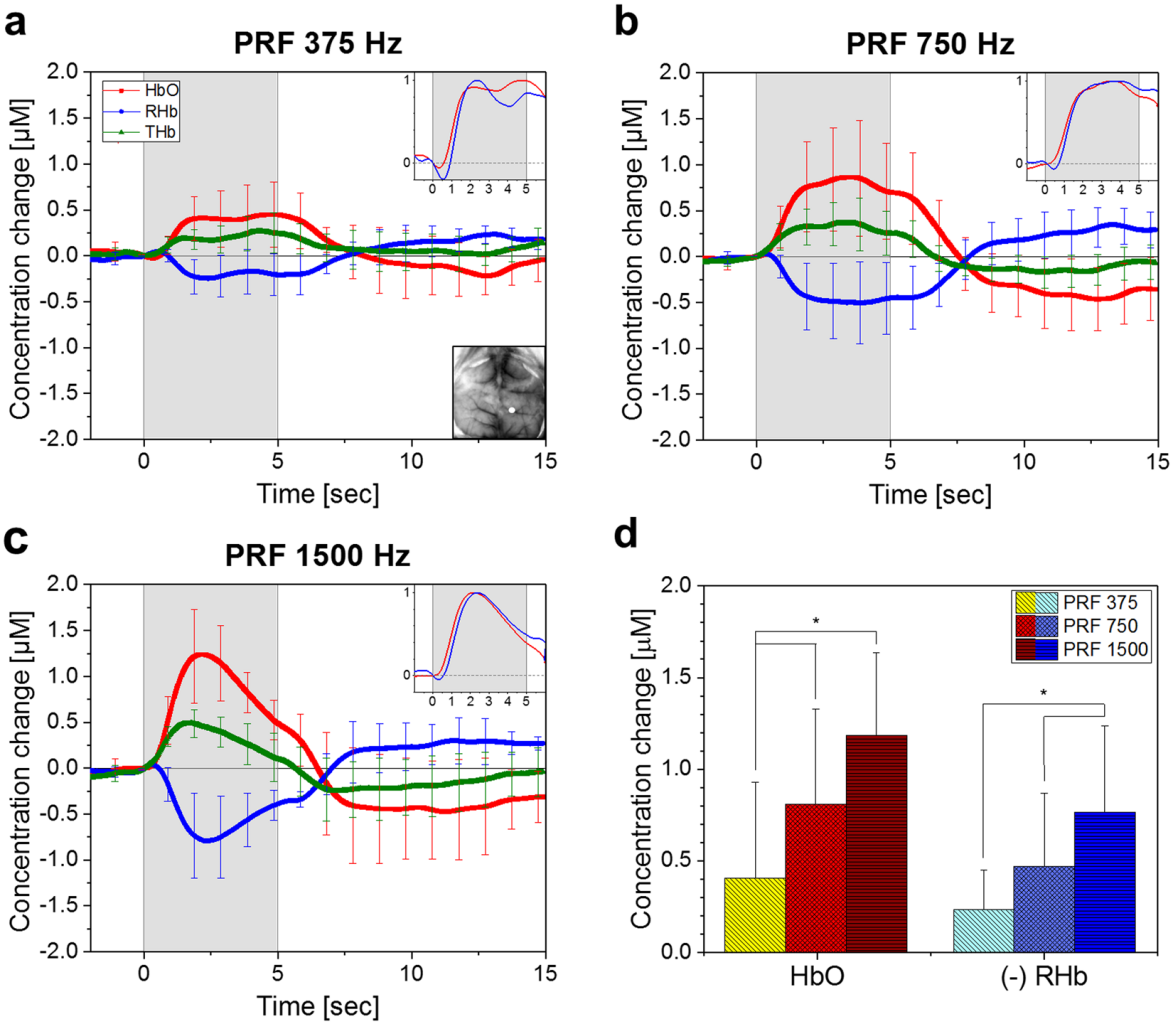
Averaged temporal hemodynamic changes from all animals. (**a**–**c**) The stimulation elicited changes in hemodynamics during and after the 5-second stimulation (gray). The time series were taken from the area indicated by the white dot (inset, **a**). The change in HbO and RHb (inset, **a**–**c**) was rescaled, after inverting RHb, to emphasize the difference in response time between the two. The hemodynamic changes during the sham condition can be found in the Supplementary Information. (**d**) Repeated measures one-way ANOVA with Tukey-Kramer post-hoc analysis (p < 0.05) of the averaged HbO, and Hb changes during 1 sec window centered at 2.7 s after stimulation onset revealed a statistically significant difference (*) in amplitude depending on PRF. In all the plots, the error bars indicate the standard deviation between the subjects.

The averaged temporal hemodynamic change across all mice in the right side below the bregma (Fig. 3a, inset) is shown in Fig. 3. The area was the same for all the mice and was chosen as it was observed to have the most pronounced amplitude change in the brain, while a prominent difference in shape or delay in phase was not observed in the hemodynamic response over the whole cortex. The increase in HbO emerged shortly after the onset of stimulation, while the corresponding decrease in RHb lags by a fraction of a second (emphasized in the inset in Fig. 3a–c, RHb was inverted) with an initial small increase in concentration reminiscent of the *initial dip* in the BOLD fMRI response^42^. The total hemoglobin concentration (THb) indicating the change in cerebral blood volume also increased after the onset of stimulation and tend to return to baseline after the stimulation.

A statistically significant difference in hemodynamics from the presented brain region was found in varying the PRF after stimulation onset over a 3 sec period (repeated measures one-way ANOVA with Tukey–Kramer *post-hoc* analysis, *p* < 0.05). The largest difference in HbO concentration change was observed at 2.7 sec after stimulation indicating the peak of HbO from PRF 1500 Hz. The averaged temporal hemodynamic response over a 1 sec window centered around the time of the peak of HbO and RHb (Fig. 3d) showed a statistically significant difference throughout the cortex.

### Decomposed hemodynamic signals

**Figure 4:**
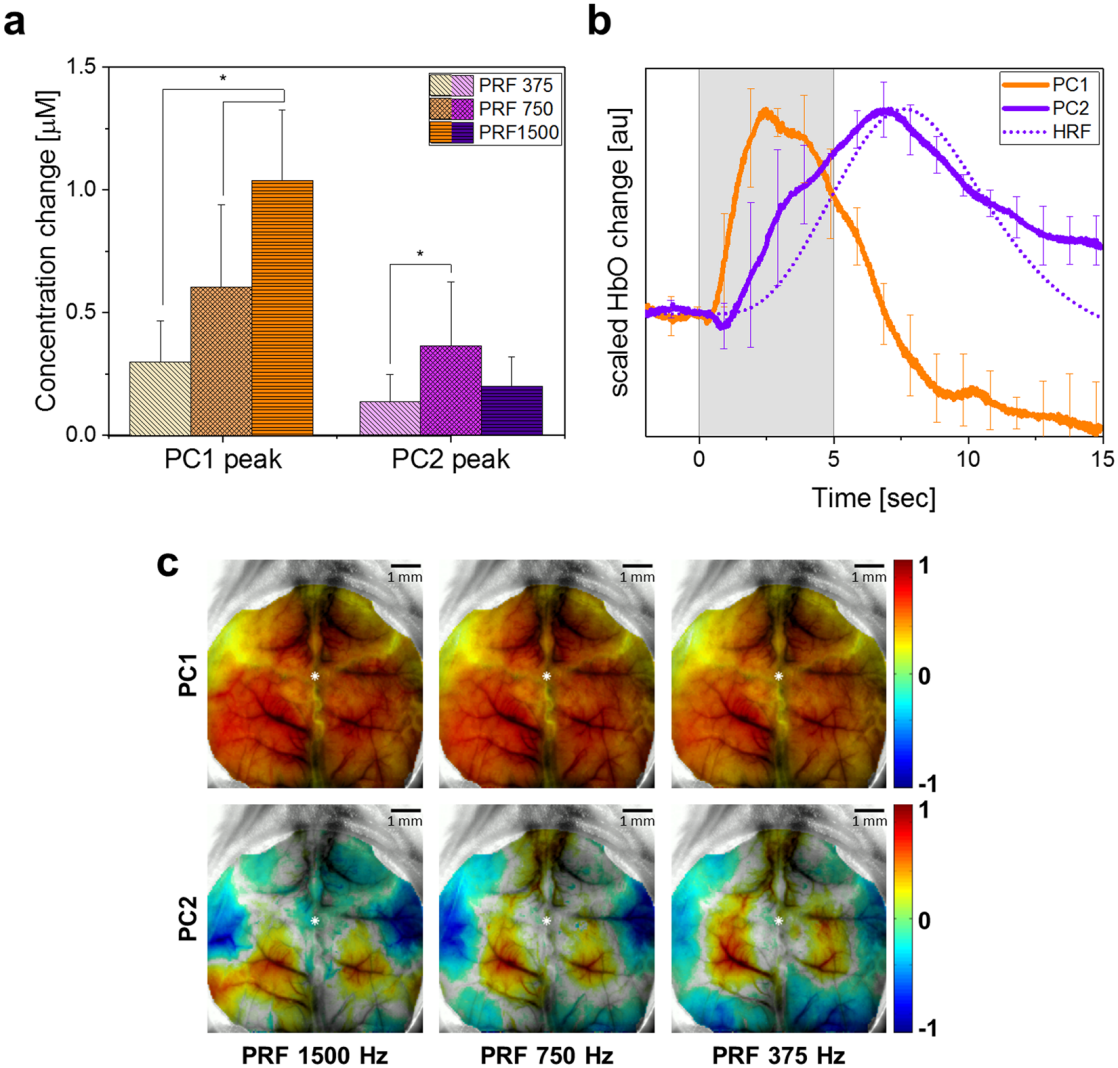
Separation of global and local hemodynamic changes using PCA. (**a**) The averaged HbO concentration change among six animals recovered from the maximum peak of PC1 and PC2. The asterisk (*) indicates the statistically significant difference (repeated measures one-way ANOVA with Tukey-Kramer post-hoc analysis; p < 0.05) in amplitude varying by tUS parameter. Error bars indicate the standard deviation between subjects. (**b**) The average of the normalized two components of the HbO signal for three different paradigms in a representative mouse. Error bars indicate the standard deviation between paradigms. The second principal component correlates strongly with the associated HRF for the stimulation. (**c**) The corresponding images from the first (top row) and second (bottom row) principal components for each paradigm. The color bar indicates the normalized magnitude of PC coefficients. The white dots indicate the bregma.

Principal component analysis (PCA), a widely-used feature extraction technique in the image processing field, was applied to separate the signals from different sources including that related to neural activity, and remove motion artefacts and other background noise. Two principal components were consistently observed from the HbO signal from six out of seven mice during all actual stimulation conditions. Similar to the original HbO signal, both components show changes initiated by tUS that have a statistically significant (repeated measures one-way ANOVA with Tukey–Kramer *post-hoc* analysis, *p* < 0.05) difference in peak amplitude depending on PRF (Fig. 4a). The decomposed signals were compared against the HRF corresponding to the given stimulation. Comparison across all tUS paradigms of the correlation coefficients between each component and the HRF does not yield a statistically significant difference (both p > 0.05); consequently, revealing a higher similarity with PC2 (*r* = 0.80 ± 0.10) than to PC1 (*r* = −0.17 ± 0.28). The average among different stimulation paradigms of the normalized time series of two components (PC1 and PC2) of a representative animal is illustrated in Fig. 4b. The topographic map of coefficients for the corresponding principal components for each paradigm for the same mouse are shown on Fig. 4c. PC1 was shown to be distributed over the whole mouse cortex, while PC2 was localized with positive and negative coefficients, where the negative coefficient indicates that the component signal is inverted, i.e. the signal decreases rather than increase.

## Discussion

To our knowledge, this is the first study utilizing OISI on an awake animal model as a neuroimaging tool to support tUS by providing maps of cerebral hemodynamic changes during sonication. The spatial map demonstrated that hemodynamic activity was distributed over the entire mouse cortex, while the time series showed the hemodynamic changes to be initiated shortly after the start of stimulation, reaching the maximum peak after 2.76 ± 0.51 s, with no changes observed during the sham stimulation.

To confirm that the observed optical changes are indeed due to cerebral hemodynamics rather than the ultrasound wave affecting the optical characteristics of the medium^43,44^, we performed additional experiment on two other mice. Considering the profound effect of anaesthesia on tUS, we performed an identical ultrasound experiment with the PRF 1500 Hz paradigm on mice under deep anaesthesia (1.8–2% isoflurane). The absence of hemodynamic changes evoked by sonication under deep anaesthesia (Supplementary Figure S2) likely excluded the concern of possible interference of acousto-optic effect with our results.

The hemodynamics under three different sonication conditions were compared with the hope to introduce OISI as a potential technique to unravel one of the main challenges in the ultrasound stimulation field, that is, to identify the optimal stimulation parameters for a desired application^45,46^. Even though the acoustic intensity within the pulse (I_SPPA_) and the total sonication time (I_SPTA_), was kept similar, our results showed a significant difference in amplitude of hemodynamic changes in the first 2.5 s after stimulation caused by the varying pulse width and repetition rate. The stimulation with the shortest pulse and highest PRF elicited the largest amplitude change of hemodynamics, while the sonication with longest pulse and lowest repetition rate induced the smallest change. The results supported the discussion of a previous work^47^ emphasizing the importance not only of the acoustic intensity itself but also of the pulse duration and repetition period. The finding might also be important for neuroprotection to minimize the effect of ischemic stroke as it relates directly to blood flow^48^.

The peak of the observed hemodynamic change was reached within the sonication period, while the typical neural activity-related hemodynamic change described by the HRF is expected to reach its maximum after the stimulation^49^. Previous neurovascular coupling reports indicated localized responses correlated to the HRF may be quenched by a dominant global response that could be extracted by blind source separation techniques^50^. The implementation of PCA revealed that the HbO change during tUS consisted of a stimulus-triggered global (PC1) and local (PC2) hemodynamic change. The global signal has a dominant influence to the original hemodynamic amplitude change, while the weaker localized response was found to be strongly correlated to the HRF. In six out of seven animals, each of these components have similar waveform regardless of stimulation condition, but statistically differing significantly in amplitude. Although the PCA-decomposed signals from the seventh mouse include the global response, the localized component correlated to the HRF was not found. While the global response can be described as a dramatic increase and gradual decrease of HbO concentration over the whole cortex, the tUS-triggered hemodynamic response likely related to neural activity was interestingly localized, and either increasing or decreasing in regions distributed symmetrically within the left and right hemispheres. The observed symmetry and activation of one region of the brain while suppressing the activity of another is consistent with previous studies^38^. The robustness of these findings was evident in the consistency of results across six mice.

**Figure 5:**
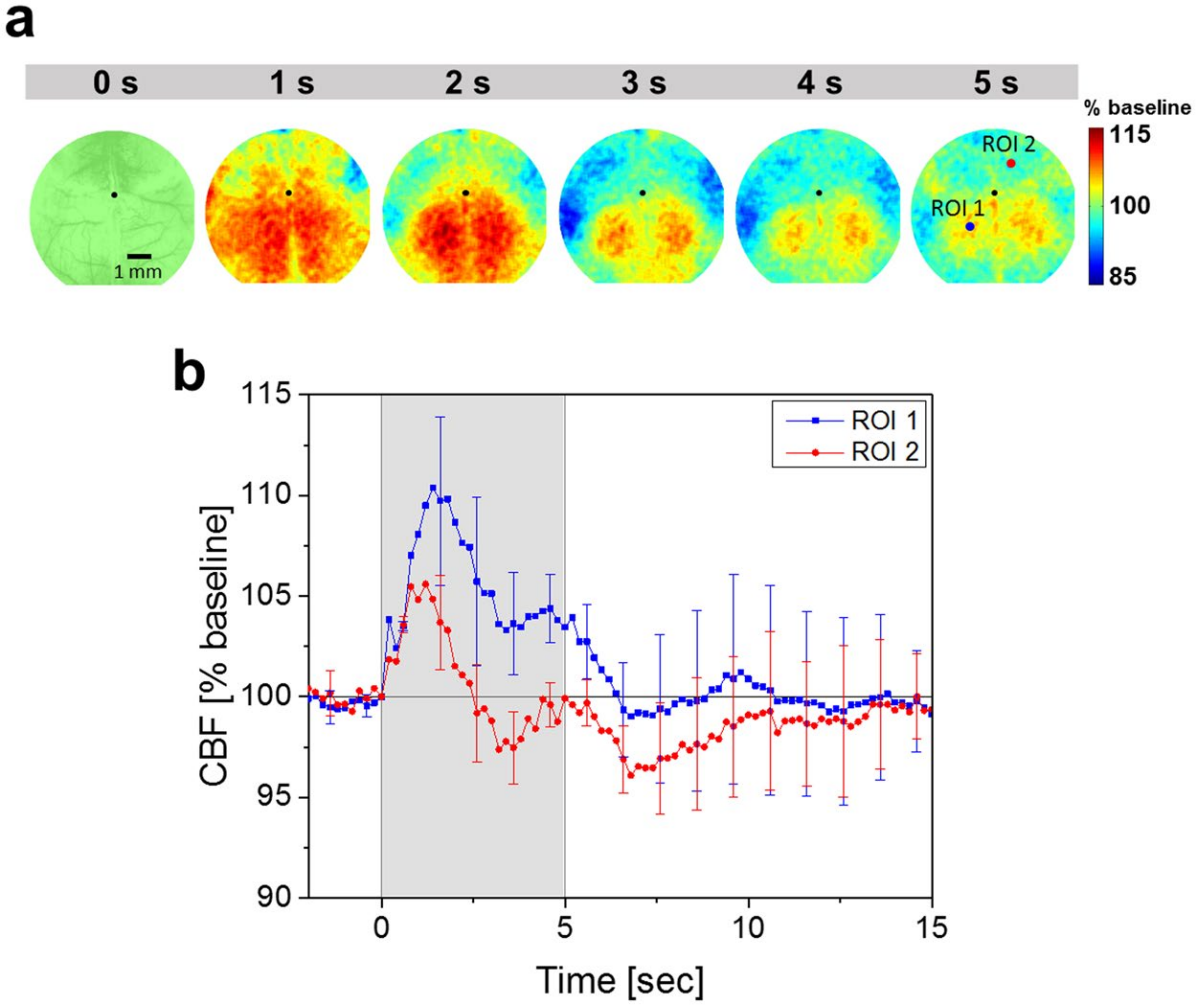
Spatiotemporal changes in CBF during tUS. (**a**) Representative spatial map of the CBF change from one mouse. An image of the brain is overlaid over the 0 s frame for reference. (**b**) Averaged temporal changes in two regions of the mouse brain (points indicated in the 5 s frame in a). The error bars indicate the standard deviation between the subjects (n = 3).

In order to support the hypothesis that the high correlation of PC2 with HRF is related to neural activity, changes in the cerebral blood flow (CBF) were measured in a separate experiment during tUS using laser speckle imaging (see Supplementary Information). The spatial map of CBF changes during sonication (Fig. 5a) shows an initial increase throughout the brain, but a CBF constantly above baseline level was observed only in the regions that overlap with the spatial map corresponding to PC2 (Fig. 4c), reinforcing the notion of neuronal activation due to tUS. The observed temporal evolution of CBF (Fig. 5b) during tUS is in agreement with a previous report^51^.

The source of the initial global increase in CBF along with the observed global hemodynamic change is still unclear. However, the absence of the global hemodynamic response in the sham stimulation and difference in amplitude depending on PRF imply that tUS might be affecting the brain in a manner other than focal neuromodulation^52^. A direct measure of neural activity concurrent with the measurement of hemodynamic change could be useful in elucidating the cause of the global response and contribute to the full understanding of the neurovascular response to tUS.

Although the ultrasound transducer was consistently placed on the right side of the head for all the mice, a stark difference in hemodynamics between the hemispheres was not observed. Considering the fact that the selected central frequency of 425 kHz, has a relatively large focal area in comparison with the mouse brain, along with the arising complexity of the acoustic pattern inside the animal skull^53^, we are not able to affirm the origin of localization and symmetry of the associated hemodynamic change. Likewise, a number of studies have demonstrated the ability of tUS to elicit a motor response even during sonication of brain regions other than the motor cortex^36,54^. We believe these observations are related to brain functional connectivity^29^, and the implementation of OISI with a higher central frequency of tUS that can provide a smaller stimulation region^52^ might be critical tools for studies in brain connectivity.

The inability to extract a highly correlated HRF component from one mouse could be related to the limitation of PCA to assume the orthogonality of the source signals. Application of an advanced blind source separation technique considering nonorthogonal sources such as independent component analysis (ICA), and extended spatial decorrelation (ESD) could be beneficial^55^. The results of PCA-decomposed RHb data were not presented due to insufficient SNR and inconsistencies in extracting the HRF-correlated signal.

In summary, OISI was shown to be a reliable neuroimaging tool providing spatiotemporal cerebral information during tUS. The results showed global and local hemodynamic response due to brain changes during sonication. A significant difference in the amplitude of the hemodynamic change was observed by varying the acoustic pulse width and repetition frequency. Thus, the technique has been shown to offer the capability to obtain insight into the effects of different tUS parameters on the brain through simultaneous measurement of cerebral hemodynamics.

## Methods

### Animal preparation

All animals were cared for in accordance with the guidelines for the Care and Use of Laboratory Animals. The protocols used in the study has been approved by the Institutional Animal Care and Use Committee (IACUC) of the Gwangju Institute of Science and Technology. A total of twelve female BALB/c mice weighing 17–20 g (9–10 weeks old) were used for the experiments (n = 7, awake; n = 2, anaesthetized; n = 3, laser speckle imaging). The number of animals was selected through the resource equation method^56^ with the intention of minimizing the sample size with enough thoroughness to accomplish the objectives of the study.

The mesoscopic wide-field cranial window for awake mouse was implemented according to the procedure described by Murphy’s group^57^. The mice were anaesthetized using an intraperitoneal injection of ketamine – xylazine cocktail (80:10 mg/kg, respectively) for cranial window surgery. The scalp fur was shaved and a midline incision was made along the top of the head and the skull was exposed. A clear dental cement and cover glass was placed on top of the intact skull to provide transparent access to brain tissue. A metal bar was glued to back of the head (4 mm posterior from bregma) to restrain the head of the awake mouse during image acquisition. Moreover, the mouse body was enclosed in a small plastic tube to restrict movement. The animals were allowed to recover for 24 hours before the actual experiment.

### Transcranial ultrasound stimulation

The tUS was achieved using a focused acoustic beam from a single element ultrasound transducer (V301-SU, Olympus Corp., Japan) operating at 425 kHz as fundamental frequency, having a low acoustic attenuation through the skull. The input signal to the transducer originated from a function generator (33220 A, Agilent, USA) magnified by a linear power amplifier (240 L, ENI Inc., USA). A 3D printed acoustic guide was attached to the end of the ultrasound transducer to provide a convenient access to the mouse head, and additionally restricting the acoustic wave propagation. A thin paraffin film (Parafilm M^®^, Sigma-Aldrich Corp, US) lined the edge of the acoustic guide to electrically insulate the mouse from the transducer. The ultrasound intensity and beam profile was measured from the tip of the waveguide using an acoustic intensity measurement system (AIMS III, ONDA, USA).

### Optical intrinsic signal imaging

The cortex was sequentially illuminated with 465 and 560 nm light using two LEDs (LED465E, Thorlabs, Inc., USA; high power white LED with optical band pass filter, FB560-10, Thorlabs, Inc., USA). The reflected light from the brain was collected using an sCMOS camera (Prime, Photometrics, USA) with an objective lens (5x Mitutoyo Plan APO, Edmund Optics Ltd, UK) at a frame rate of 66 Hz. The camera exposure was synchronized with the LEDs using a built-in multi-trigger output function from the camera which gives a final sampling rate of 33 Hz for each light source, and image size of 1024 × 1024 with a spatial resolution of 7.1 µm per pixel.

### Experimental design

Each animal was initially anaesthetized with 3% isoflurane to restrain the animal in the stereotaxic frame. Once fixed, the animal is given at least 30 minutes to recover from anaesthesia before the start of data acquisition. All animals received four different stimulation conditions, including sham, within a single image acquisition experiment. The stimulation paradigms were selected in a way to keep the same spatial peak pulse average (I_SPPA_ = 1.84 W/cm^2^) as well as spatial peak time average intensity (I_SPTA_ = 129 mW/cm^2^) but with different pulse durations and repetition rates. The sonication parameters selected are considered safe and below the limits set by the US Food and Drug Administration (FDA) for diagnostic ultrasound imaging (I_SPTA_ of 720 mW/cm^2^, I_SPPA_ of 190 W/cm^2^, MI of 1.9)^58^. Furthermore, the maximum peak negative pressure of 0.53 MPa used in the study is far below the threshold for thermal effects or cavitation related brain tissue damage^59^.

The experiment on the awake animals consisted of 100 randomly shuffled trials, with 25 trials for each paradigm. Each trial image acquisition lasts for a total of 17 s including 5 s of stimulation containing ten ultrasound pulse trains with a repetition rate of 2 Hz. The interval between the beginning of each trial was fixed to 1 minute. The ultrasound was applied non-invasively to the brain through an acoustic guide filled with gel coupled to the skin on the right side of the mouse head, avoiding the whisker pad, and directed towards the bregma to provide a full image of the cortical brain. The gel provides a 2 mm distance between the skin and the edge of the guide. Sham trials were applied using the same protocol and setup as stimulation trials but without any input to the transducer.

The anaesthetized measurements were performed with an identical protocol as in the awake animal experiments but applying only the PRF 1500 Hz stimulation. A heating pad was used to maintain the mouse body temperature while under anaesthesia.

### Signal processing

The original images were resized to 128 × 128 using the Image Processing Toolbox from Matlab (Mathworks, Inc., USA) to decrease shot noise and to avoid overfitting for further data analysis. The time series analysis begins by converting the image intensity from each wavelength into relative concentration changes of HbO and RHb using the modified Beer-Lambert law^60^, along with a 1D zero-phase low pass filter applied at 1 Hz to reduce the higher frequency respiratory and cardiac noises. The total hemoglobin (THb) change was obtained by summation of HbO and RHb. The results of HbO and RHb of the 25 trials from each paradigm were averaged to increase SNR.

In addition, principal component analysis (PCA) (Statistics and Machine Learning Toolbox, Matlab, Mathworks Inc., USA) was applied to remove motion artefacts and decompose the signal into different sources^61^. One-way ANOVA with Tukey-Kramer post-hoc analysis (α = 0.05) was performed to compare hemodynamic response within and (repeated measures) between mice. The averaged signal over the whole cortex from 25 individual trials were used for the statistical analysis within mice, while the averaged hemodynamics over all trials from the indicated cortical area from all the mice were utilized for the between-group analysis.

## Electronic supplementary material

Spatiotemporal hemodynamic response during transcranial ultrasound stimulation

supplementary information
