# Supplementary material for: Monitoring cerebral hemodynamic change during transcranial ultrasound stimulation using optical intrinsic signal imaging
Source: Sci Rep. 2017 Oct 13;7:13148. doi: 10.1038/s41598-017-13572-0 (PMC5640689; doi:10.1038/s41598-017-13572-0)
Supplement: Supplementary file 2 — supplementary information [file 41598_2017_13572_MOESM2_ESM.pdf]

Monitoring cerebral hemodynamic change during transcranial ultrasound stimulation using optical intrinsic signal imaging

Evgenii Kim[1], Eloise Anguluan[2], Jae Gwan Kim[1, 2, *]

[1] School of Electrical Engineering and Computer Science, Gwangju Institute of Science and Technology, Gwangju 61005, Korea
[2] Department of Biomedical Science and Engineering, Gwangju Institute of Science and Technology, Gwangju 61005, Korea
[*] jaekim@gist.ac.kr

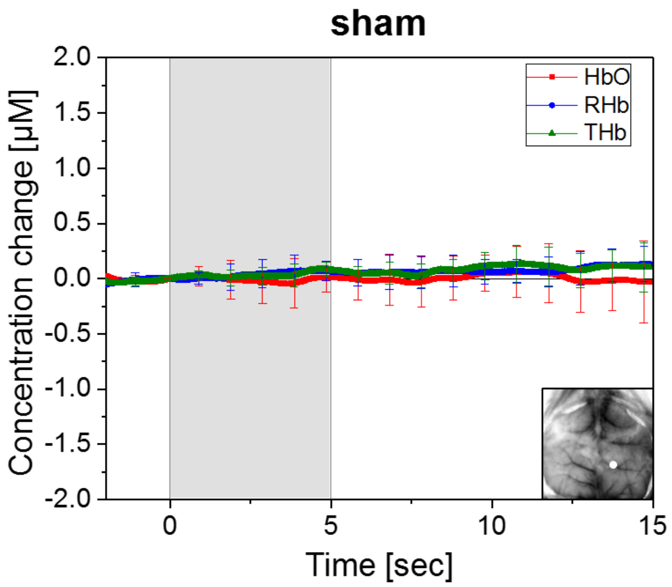

Supplementary Figure S1. Averaged temporal hemodynamic changes from all animals during sham condtion (gray). The time series were taken from the area indicated by the white dot (inset).

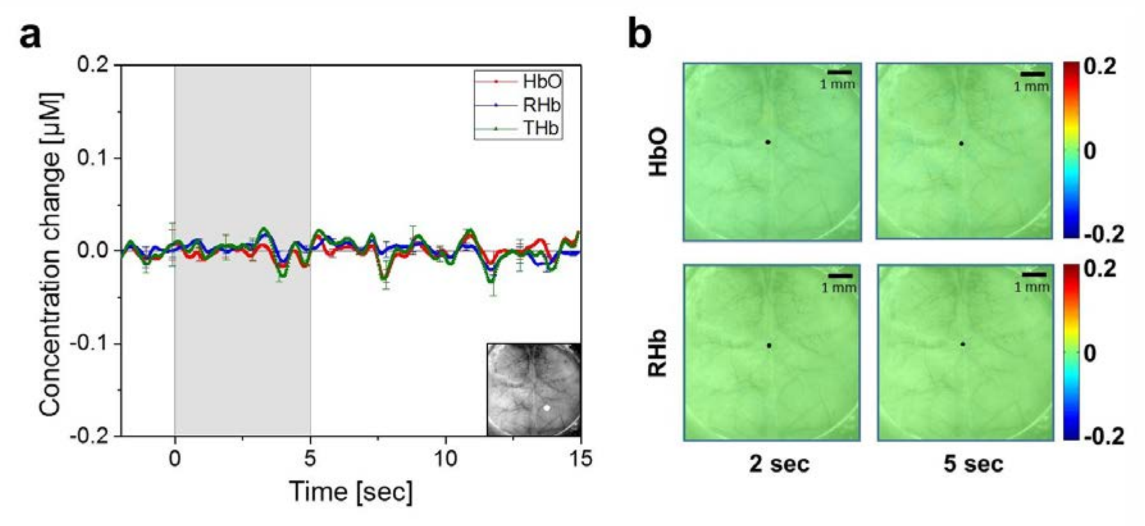

Supplementary Figure S2. Absence of spatiotemporal hemodynamics response during tUS for a deeply anaesthetized mouse. (a) The averaged temporal hemodynamic change from two mice during stimulation from the area indicated by the white dot in the inset. Error bars indicate the standard deviation between the two subjects. (b) Spatial maps of cerebral hemodynamic change during tUS for a representative isoflurane-anaesthetized mouse at 2 s and 5 s after stimulation. The color bar indicates the concentration change in μM. An image of the mouse cortex taken at 560 nm is overlaid on the frames for spatial reference. The black dots indicate the bregma.

Laser speckle imaging

Cerebral blood flow during ultrasound stimulation were monitored using LSI (Supplementary Fig. S3) separate from OISI experiments but using the same ultrasound stimulation protocol for PRF 1500 Hz at 50 trials for each subject. The mouse cortex was illuminated by a 660 nm expanded laser beam (LDCU5/A510, Power Technology, Inc., USA). Specular reflections were minimized by placing a crossed linear polarizer in front of the collection optics. The resulting speckle field was imaged on an sCMOS camera (pco.edge 4.2, PCO, Germany) at 100 Hz with 5 ms exposure time. In all LSI experiments, the eyes of the mice (N = 3) were covered to prevent exposure to the laser.

The speckle images were analyzed in the temporal domain for each trial with a time window of 20 frames[1]. The CBF images obtained from 50 trials were then averaged for each animal. To obtain the spatial maps of CBF change, the computed CBF were resized and filtered using a 2-dimensional averaging filter with a 4x4 window.

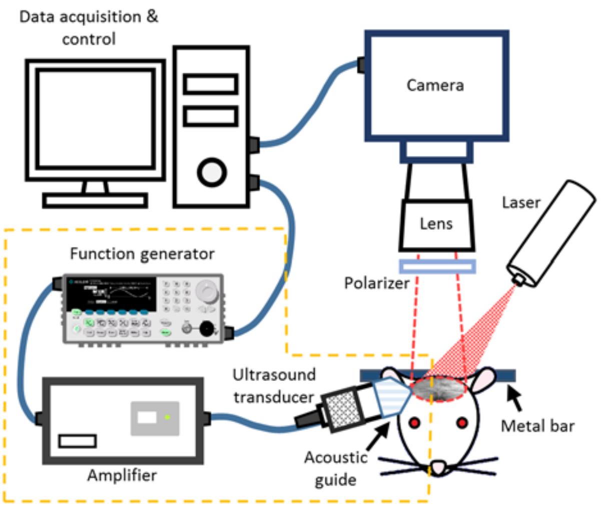

Supplementary Figure S3. Laser speckle imaging (LSI) setup. The ultrasound stimulation during LSI is the same as PRF 1500 Hz for the OISI experiments.

1. Cheng, H. *et al*. Temporal statistical analysis of laser speckle images and its application to retinal blood-flow imaging. *Opt. Express* **16,** 17525–17530 (2008).
